# Novel Reproducible Manufacturing and Reversible Sealing Method for Microfluidic Devices

**DOI:** 10.3390/mi13050650

**Published:** 2022-04-19

**Authors:** Camilo Pérez-Sosa, Ana Belén Peñaherrera-Pazmiño, Gustavo Rosero, Natalia Bourguignon, Aparna Aravelli, Shekhar Bhansali, Maximiliano Sebastian Pérez, Betiana Lerner

**Affiliations:** 1 IREN Center, National Technological University, Buenos Aires B1706EAH, Argentina; camilo.bm91@gmail.com (C.P.-S.); anibelenp@hotmail.com (A.B.P.-P.); gustavorosero@gmail.com (G.R.); nbourgui@fiu.edu (N.B.); 2 Department of Electrical and Computer Engineering, Florida International University, Miami, FL 33174, USA; sbhansa@fiu.edu; 3 Applied Research Center, Florida International University, Miami, FL 33174, USA; aaravell@fiu.edu; 4 Faculty of Engineering, University of Buenos Aires (FIUBA), Buenos Aires C1063ACC, Argentina

**Keywords:** reusable microdevice, reversible sealing, PMMA plates, microdroplets

## Abstract

Conventional manufacturing methods for polydimethylsiloxane (PDMS)-based microdevices require multiple steps and elements that increase cost and production time. Also, these PDMS microdevices are mostly limited to single use, and it is difficult to recover the contents inside the microchannels or perform advanced microscopy visualization due to their irreversible sealing method. Herein, we developed a novel manufacturing method based on polymethylmethacrylate (PMMA) plates adjusted using a mechanical pressure-based system. One conformation of the PMMA plate assembly system allows the reproducible manufacture of PDMS replicas, reducing the cost since a precise amount of PDMS is used, and the PDMS replicas show uniform dimensions. A second form of assembling the PMMA plates permits pressure-based sealing of the PDMS layer with a glass base. By reversibly sealing the microdevice without using plasma for bonding, we achieve chip on/off configurations, which allow the user to open and close the device and reuse it in an easy-to-use way. No deformation was observed on the structures of the PDMS microchannels when a range of 10 to 18 kPa pressure was applied using the technique. Furthermore, the functionality of the proposed system was successfully validated by the generation of microdroplets with reused microdevices via three repetitions.

## 1. Introduction

Microfluidics refers to the science that allows the study and manipulation of fluids on the micrometer scale [1]. Over the last two decades, microfluidic systems have been established as promising platforms for many lab-on-chip (LOC) applications in several research fields, such as chemistry, biology, medicine, and engineering. The main advantages of the microfluidic approach lie in the ability to control liquids under a laminar regime, reduction in the amount of reagents and sample volume, shorter analysis time, reduction in cost, and portability [2]. Polydimethylsiloxane (PDMS) is the most used material for the fabrication of microfluidic devices as it is optically transparent, biocompatible, easy to fabricate and its manufacture does not require high capital investment or cleanroom conditions [3]. One key step for PDMS chip manufacturing is to bond the PDMS replica with another substrate (usually glass) to assemble the microfluidic channels and possess high bond strength and stability. An extensive and critical review published by [4] gathers many methodologies for irreversible or reversible PDMS sealing to PDMS or other different planar structures—such as silicon, glass, or other polymers—and different ways to make fluidic or electrical connections. Among all sealing methods described to date, oxidation using oxygen plasma treatment is the most common method [5]. Although irreversible sealing methodologies are useful due to their strong bonding and ability to support high pressures, they present some disadvantages, such as low reusability and difficult internal accessibility into microchannels.

On the other hand, the reversible sealing of microdevices allows easy disassembling, cleaning, reassembling, and reduction of manufacturing time. PDMS chips can directly attach to other PDMS or glass slides through Vander Waals forces. For example, Park et.al. [6] fabricated microfluidic culture platforms using reversible bonding by lightly touching the PDMS on a glass coverslip previously coated with poly-L-lysine [6]. However, this procedure is only suitable for low pressures (less than 35 kPa), and chips are prone to debonding or leaking. To overcome this drawback, other techniques with the ability to support higher pressures (up to 100 kPa) have been proposed. For example, sealing by vacuum suction [7,8,9] consists of crossing a channel air network around the main microchannels where the vacuum is applied by aspiration. Nevertheless, this application requires additional working space for the vacuum source, as well as an additional microchannel network around the main microdevice. A magnetic seal is another technique proposed for reversible bonding by applying an external and controlled magnetic force [10,11]. More recently, Tsao and Lee [12] fabricated an iron oxide magnetic microparticle PDMS composite material, offering the possibility to cast in an opaque-view or a clear-view. However, with the opaque-view material, optical detection was not possible, and the surface roughness increased with respect to native PDMS [13]. In general, reversible sealing by magnetic forces allows a uniform and long-range of pressures depending on the applied magnetic field strength. Nevertheless, the presence of slab-shaped magnetic material restricts the device microchannel network to very simple geometries [12]. Commercial adhesive tapes for the reversible bonding of microfluidic chips have also been reported, where the tape is placed under a PDMS chip and baked at 65 °C for 2 h. The tape can be peeled off, and the microdevice can be reused after it has been washed; in contrast, adhesives increase the possibility of contamination [12].

In the current paper, we present a novel modular microfluidic platform that is easy to assemble and allows the reversible bonding and reuse of the microfluidic device. This innovative and mechanical approach for reversible sealing in a microfluidic device consists of placing the polydimethylsiloxane (PDMS) chip between two planar poly(methyl methacrylate) (PMMA) plates and applying mechanical and uniform pressure. The reversible seal proposed in this work is mechanical clamping, a technique that has been explored in general for glass–PDMS–glass sandwich [14]. In addition, a molding method is shown to manufacture microfluidic devices by casting PDMS in a reproducible and safe way (REPSAF). This method also uses less PDMS and produces polished walls.

As a proof of concept, we validated the method by applying PDMS chips for droplet generation. Droplets are produced by breaking the surface tension between a continuous phase (oil) and a dispersed phase (water) within multiple models of devices that allow controlling flow mixtures and droplet size [15,16]. However, a set-up in many of the applications requires many devices, which implies great expenditure in materials and manufacturing time. We address this issue with the chip on/off configuration to enable reusability obtained with the new methodology.

## 2. Materials and Methods

### 2.1. Mechanical Pressure Set-Up

**Figure 1:**
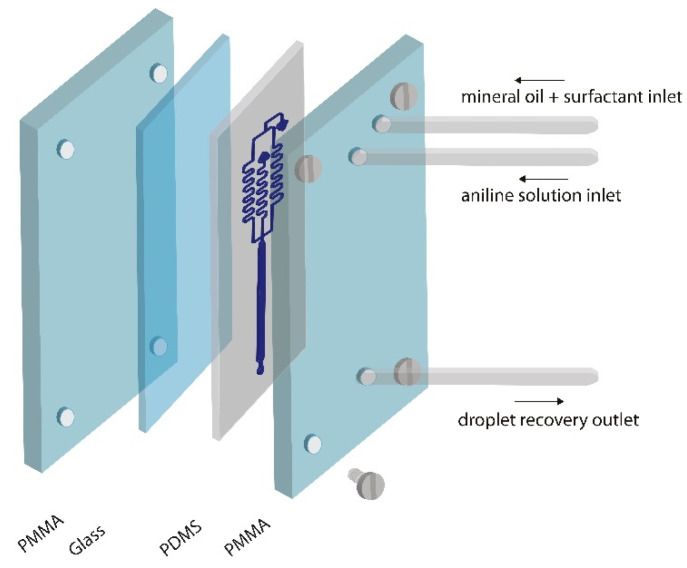
Assembled set-up scheme for microfluidic experiments.

The mechanical pressure assembly consists of two PMMA plates (PLEXIGLAS^®^).-The supplier uses a manufacturing protocol combining bulk emulsion and solution emulsion). The plates were made to measure corresponding to a base and a lid, with dimensions of 5 × 85 × 70 mm. Figure 1 shows how the microchannels containing PDMS and the glass base of the chip are placed between the PMMA plates. Four JM ^®^ brand stainless steel screws of same size were used to generate the same pressure (D for certain experiments: 8.16 mm and L: 17 mm) The screws were fixed to reversibly join the microdevice and apply mechanical pressure. The screws are located at 50 mm in width and 55 mm in length from each other. The PMMA top plate has one inlet hole and one outlet hole into which 24 mm long and 3 mm wide polytetrafluoroethylene (PTFE) hollow screws are inserted and then connected to polyvinyl chloride (PVC) tubing 18 mm long and 3 mm wide. These tubes are connected to a syringe pump (ADOX 22a)

### 2.2. Design and Fabrication of Microfluidic Device

**Figure 2:**
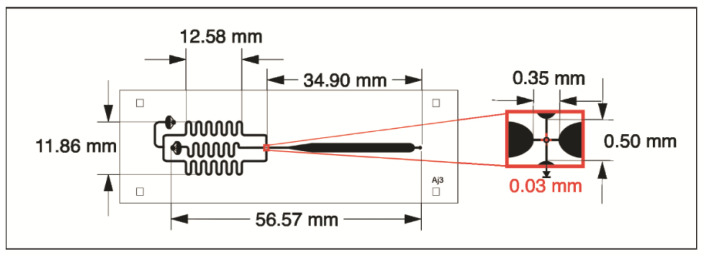
Droplet-forming microdevice design.

The droplet-forming microdevice (Figure 2) was photolithographed in a high relief mold with the desired pattern on a 700 µm thick silicon wafer (Virginia Semiconductor, Inc., Fredericksburg, VA, USA), using the negative resin SU-8 (MicroChem, Round Rock, TX, USA). The microchannels have a final height of 150 µm. Next, the mold was placed under vacuum with trichloro (1H, 1H, 2H, 2H-perfluoro-octyl) silane (Sigma, St. Louis, MO, USA) for 1 h to protect the SU-8 resin from detachment by releasing PDMS from the mold. The PDMS was mixed with the curing agent in a 10:1 ratio and the mixture was placed under vacuum for 1 h to remove air bubbles. Next, the mixture was poured back under vacuum for 1 h and cured in an oven at 70 °C for 70 min. The PDMS was molded, and the fluidic connection ports were constructed by drilling holes in the PDMS with a hole punch (21-gauge, internal diameter of 0.51 mm). Finally, the PDMS device was assembled with a glass base.

### 2.3. Deformation and Flow-Rate Measurement

Testing and validation of the newly manufactured microdevice has been conducted for changes in the structure (channels) and the flowrates at various pressures. Channel deformation produced by compression has been measured with a stereomicroscope (BioTraza, Zhejiang, China) 2,3 × magnification. The stereomicroscope has been coupled to a Canon EOS 600D camera HD (1080p 1920 × 1080) recording at 29.97, 25, or 23.976 frames/s. The deformation is determined by taking serial images at different pressures focused on a single design structure. Pressure on the microfluidic device was measured with a force-sensing resistor (Interlink Electronics FSRTM 400—Interlink Electronics, Lake Forest, IL, USA) coupled to a multimeter UNI-T (UT39A) located at the right corner below the chip. ImageJ software [17] was used for measuring structure length (pixels) changes at different pressures.

Flow rates at different pressures were measured using aqueous suspensions of Acid Blue 1 dye 0.648 mg mL^−1^ (Sigma-Aldrich, St. Louis, MO, USA). Video images were analyzed to measure the time employed by the front of the dye to travel a fixed microchannel distance.

### 2.4. Droplet Generation

A flow-centered emulsion droplet microfluidic device was designed, constructed, and used for the generation of monodisperse micrometric-sized droplets, consisting of two inlets and one outlet for droplet recovering. The internal phase, 2% blue aniline solution (% *w/v*) was pumped (AcTIVA Infusion ADOX A22, CABA, Argentina) at a constant rate of 0.80 µL/min, the continuous phase mineral oil (Sigma-Aldrich, St. Louis, MO, USA) with SPAN 80 surfactant (Sigma-Aldrich) (5% *w/v*) was pumped at a rate of 1.15 µL/min.

### 2.5. Device Reuse

To achieve optimal reuse of the device, a method based on continuous washing is proposed. In an airtight container, the device was immersed in a solution containing a commercial degreaser (CLEAN LAB^®^) for 10 min, and then this device was rinsed with distilled water. The next step included immediate immersion of the device in a new commercial degreaser solution for another 10 min, followed by a new washing with distilled water. Later, the device was immersed in absolute ethanol (96%) for 10 min in a new airtight container, followed by a distilled water rinse. The device was immersed again in 70% ethanol for another 10 min. Finally, this device was rinsed with Milly Q quality water and subjected to heat (70 °C) for 2 h using Thermo Electron Precision oven.

### 2.6. Droplet Images

The Olympus BX40 microscope with 10X lenses and a Canon EOS 600D digital camera attached to the microscope was brought out to view and acquire video of drop formation. Images were obtained from a stack of multiple microscope acquisitions on the surface of the device. To analyze the size distribution of the produced droplets, the area occupied by 100 drops in each experiment was subsequently measured using image J processing software with the specific plugin to visualize circular objects [18]. Additionally, the average droplets diameter and standard deviation was reported.

## 3. Results and Discussion

### 3.1. Reproducible and Secure Microdevice Fabrication

Pressure needs to be uniform throughout the device that makes the devices reusable and work properly. To achieve uniform pressure, it is essential that the devices being used always have exactly the same height, length, and width dimensions. The traditional system commonly used for the manufacture of PDMS devices consists of cutting the PDMS deposited on the mold (usually a silicon wafer) with a scalpel. This method is not reproducible and has a user error that prevents the obtaining of two different chips with the same measurements. Furthermore, the method is very risky, since the cutting tool tends to slip out of the guide and can cause damage to the user’s hand, either in the silicon mold or directly in the micro-device.

**Figure 3:**
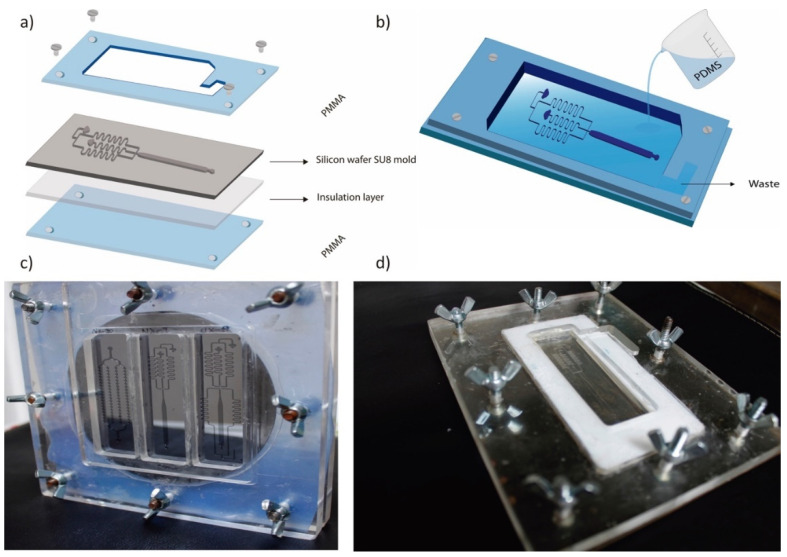
PDMS chip fabrication scheme. (**a**) set-up element description. (**b**) PDMS pouring onto SU-8 mold located between PMMA plates provided with a waste chamber. (**c**) Frontal view of the assembled set-up with the SU-8 silicon wafer. (**d**) Lateral view of cured PDMS slab ready to be removed from the mold.

To manufacture reproducible devices in a safe way, the current PMMA system was designed and manufactured as shown in in Figure 3. Using this method, it is possible to produce devices that have exact dimensions in height, width, and length.

First, the silicon wafer is placed between the PMMA set-up and an insulation layer (Figure 3a). Then, PDMS is poured into the assembled PMMA set-up provided with a waste chamber (Figure 3b). The upper PMMA plate contains a cavity of 3 mm height, 2 mm width, and 5 mm depth, corresponding to the required dimensions of the PDMS chip. This system includes a place for the PDMS excess; therefore, reproducible chips of 5 mm height can be fabricated, in contrast to PDMS chip fabrication directly over silicon wafers, where the chip height is very difficult to be controlled because it depends on the amount of added PDMS. Figure 3c shows the SU-8 silicon wafer between the two PMMA plates and Figure 3d presents the cured PDMS slab with the microchannels ready to be removed from the mold.

**Figure 4:**
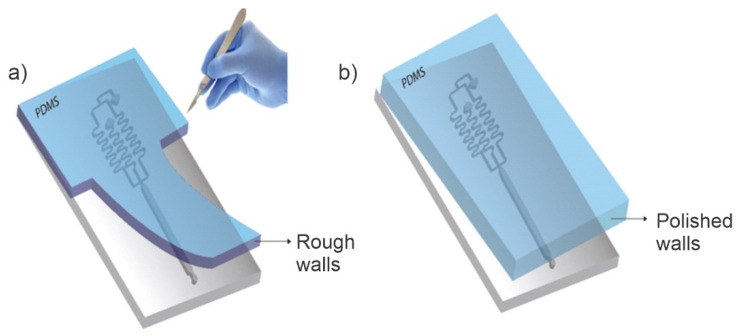
Stripping of the PDMS slab. (**a**) Traditional methodology, the user cuts it manually with the scalpel. (**b**) The reproducible and safe (REPSAF) methodology proposed in this work.

After curing, using the reproducible and safe (REPSAF) method, the fabricated devices are safely removed from the mold by unscrewing the four screws shown in Figure 4. This method avoids the cutting risk caused by the traditional method. The REPSAF methodology also has other advantages over traditional PDMS microdevices fabrication, such as (1) the chip walls become transparent, contrary to the opacity and roughness produced by a PDMS cut with a razor blade (Figure 4); and (2) the wastage of PDMS material is much lower than the one produced when the chip is fabricated with silicon wafer (around 30–50% more efficient).

### 3.2. Chip Deformation Measurements

**Figure 5:**
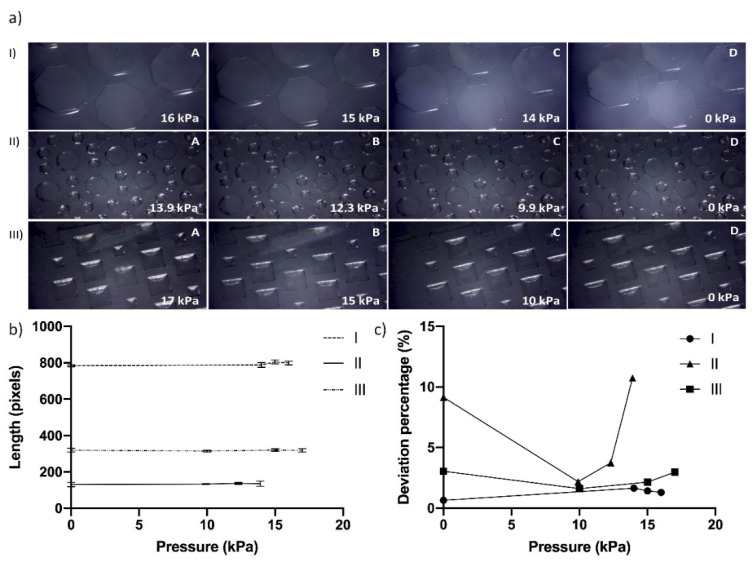
(**a**) Images of different PDMS microfluidic devices (**aI**–**aIII)** by applying different pressures. Changes in the morphology and the structure size were analyzed under high (**A**), intermediate (**B**), low (**C**), and no pressure (**D**). Four measurements at each pressure were obtained for each image. (**b**) Structure lengths (pixels) of different microfluidic devices (I, II, and III) measured according to the applied pressure (kPa) Error bars represent standard deviation between measurements. (**c**) Variation of deviation percentage in length measurements for each applied pressure.

PDMS microfluidic devices containing hexagonal micropillars (Figure 5(aI,aII) and square-shaped cavity (Figure 5(aIII)) structures were employed to measure deformation. The microdevices were exposed under several pressures in the range of 0 to 18 kPa. As shown in Figure 5, the length in pixels of the microstructures (hexagonal micropillars and square shaped cavity) was measured from the higher to the lower pressure exposition by taking serial images, to evaluate microstructures deformation.

Image analysis results showed no changes in the structure of the microchannel between the maximum, intermediate, or minimum pressure applied on any of the analyzed chip designs (Figure 5(aI–aIII)).

As observed in Figure 5b, the variation of length measurements of the same structure at different pressures is not significant in the three microdevices. In Figure 5c, the deviation percentage was calculated for each microdevice. Microdevices I and III showed a deviation percentage lower than 3%, whereas microdevice II presented a deviation percentage close to 11%. It could be due to its structures (pillars) being smaller and resulting in less accurate measurements. If there is no applied pressure (0 kPa), the measured deviation is not 0 due to the fact that the smaller the structure, the higher error. However, for Microdevice I, the measured deviation is 0.64% as the structures are larger.

### 3.3. Droplet Production Results

The reusability of the microdevice fabricated with the RESAF method and assembled using the PMMA plates system was demonstrated with the reproducibility of monodisperse droplets. Droplets were produced and measured in three different experiments (replica) using the same microfluidic device; droplet size ranged from 69.77 to 80.21 µm. A constant flow of 0.80 µL/min of the disperse phase and 1.15 µL/min for the continuous phase were used to obtain droplets with the mentioned size.

**Figure 6:**
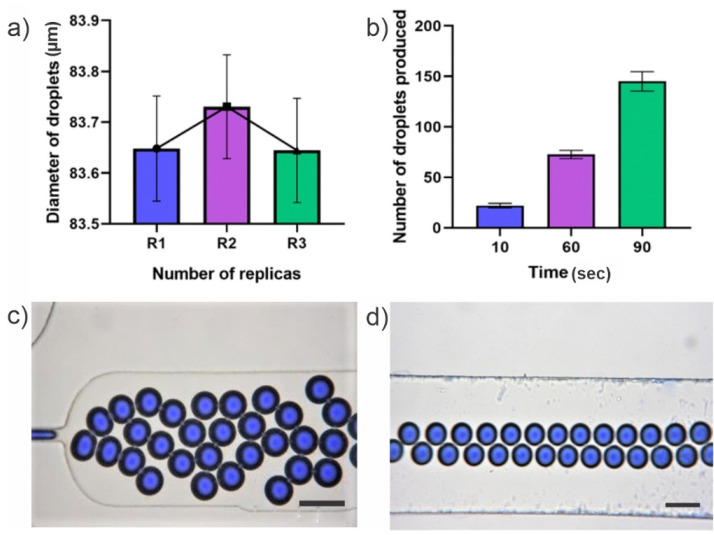
Droplet size and number of droplets produced. (**a**) Size of droplets produced after using the same microdevice three times (two reuses). (**b**) The average number of droplets produced in a time window measured in the output channel in the three assays of reuse. (**c**) Representative image of the droplets produced in the flow encounter model. (**d**) Representative image of the droplets produced in the outlet channel. Scale bar, 100 µm.

After producing the droplets, the device was disassembled, un-tacked from the PMMA set-up (shown in Figure 1), and washed as indicated in the Section 2.5. Afterwards, it was reassembled in the same set-up. Figure 6a. shows the size of microdroplet formed after the repeated washing and reuse processes. The results show that neither drops nor the device have suffered variations with the different uses.

Additionally, the average number of droplets produced by the microdevice (for the three replicas) was calculated, in a temporary space of 10, 30, and 90 s as shown in Figure 6b. With this information, we measured the performance of the device after the cleaning cycle and reuse. As a result, the device performance did not decrease, which confirms that our system is reusable and easy to use.

## 4. Conclusions

In this work, a reversible and low-cost microfluidic sealing methodology is proposed. It consists of mechanically holding the chip between two PMMA plates. This proposed methodology does not require clean room facilities for device cleaning, nor does it require plasma bonding, and it is easy to perform. Furthermore, the proposed manufacturing method allows simple unmolding, as well as uniform dimensions throughout the entire PDMS chip.

In addition, it is possible to save a considerable amount of PDMS, since the methodology limits waste due to its closed edges for each model. The methodology increases the versatility of the use of microfluidic devices since it does not imply an irreversible union.

In our work, we verified the reliability and performance of the droplet-forming microdevice that has multiple uses, such as the recovery of droplets with chemical compounds, drugs, the recovery of specific clone cells, and hydrogels for long-term culture.

It is also important to note that the REPSAF system not only applies to droplet-forming microdevices, but it can also be used with cell culture chips, diluters, and applications such as EOR assisted oil recovery. In addition, the system can be used with different materials such as glass, plastic, or PMMA for several applications.
